# Advances in the Design of Phenylboronic Acid-Based Glucose-Sensitive Hydrogels

**DOI:** 10.3390/polym15030582

**Published:** 2023-01-23

**Authors:** Simona Morariu

**Affiliations:** “Petru Poni” Institute of Macromolecular Chemistry, Grigore Ghica Voda Alley 41A, 700487 Iasi, Romania; smorariu@icmpp.ro

**Keywords:** hydrogel, self-healing, glucose sensitivity, insulin releasing, phenylboronic acid, dynamic covalent chemistry

## Abstract

Diabetes, characterized by an uncontrolled blood glucose level, is the main cause of blindness, heart attack, stroke, and lower limb amputation. Glucose-sensitive hydrogels able to release hypoglycemic drugs (such as insulin) as a response to the increase of the glucose level are of interest for researchers, considering the large number of diabetes patients in the world (537 million in 2021, reported by the International Diabetes Federation). Considering the current growth, it is estimated that, up to 2045, the number of people with diabetes will increase to 783 million. The present work reviews the recent developments on the hydrogels based on phenylboronic acid and its derivatives, with sensitivity to glucose, which can be suitable candidates for the design of insulin delivery systems. After a brief presentation of the dynamic covalent bonds, the design of glucose-responsive hydrogels, the mechanism by which the hypoglycemic drug release is achieved, and their self-healing capacity are presented and discussed. Finally, the conclusions and the main aspects that should be addressed in future research are shown.

## 1. Introduction

In the last years, many studies have been directed towards the formulation of gels (hydrogels, nanogels, microgels) based on polymers due to their application potential in various fields, such as cosmetics [1], pharmaceuticals [2], biomedical [3], food [4], biotechnology [5], etc. Polysaccharides-based hydrogels characterized by biocompatibility, biodegradability, and non-toxicity can be applied in various biomedical interface applications. The presence of cellulose, chitosan, alginate, or starch into hydrogels gives the properties required for their application as a wound dressing (diffusion control, constant temperature and adequate humidity maintaining, gas exchange between the wound and the outside, etc.) [6]. Nano- and microgels have proven to be much more effective in the biomedical applications due to their faster response to external stimuli [7]. By incorporation of polysaccharides, nano- and microgels can be used as components in nutraceuticals and food as well as drug and protein carriers in the treatment of cancer or rheumatoid arthritis and in tissue regeneration [8].

The large number and continuous increase of diabetics have led to the orientation of a lot of research towards finding new high-performance sensors for glucose level monitoring. Thereby, some non-invasive techniques of glucose measurement (continuously or intermittent) by using different fluids from the body (i.e., tears, sweat, saliva, urine, interstitial fluid, blood) have been developed [9,10,11]. The hydrogels integration in various devices has been considered due to their ability to retain water (also allowing the diffusion of different compounds), and possibility to design their structure so that they respond to different external stimuli: temperature, pH, enzymes, glucose, electric and magnetic field [12,13].

Owing to the possibility to configure the properties by changing their structure, hydrogels have opened new opportunities in the development of high-performance technologies and devices with high sensitivity and accuracy for detecting and treating diabetes. Conductive hydrogels have become promising candidates for the development of electronic devices, such as sensors, actuators, soft electronics or bioelectronic devices, due to their biocompatibility, electrical responsiveness, and biomimetic features [14,15,16,17].

In the last decades, there have been major advances in the field of designing devices for glucose detection and insulin delivery for the treatment of diabetes. Polymer hydrogel-metallic nanoparticles nanostructures, with unique optical properties, represent an attractive class of materials with plasmonic characteristics suitable for the development of smart biosensors [18]. The biodetection is quantified by the changes of the optical properties of plasmonic nanostructures because of a physical stimulus or a specific interaction with the molecules from the analyzed liquid [19]. Ziai et al. [20] developed a glucose-sensing system, inspired by the structure and optical properties of the chameleon skin, consisting of a layer of poly(ethylene oxide)/poly(caprolactone) between two layers of hydrogel based on poly(*N*,*N*-isopropylacrylamide-*co*-*N*-isopropylomethacrylamide) with included silver nanocubes. This sensor used a non-invasive method to detect glucose from urine and exhibited antibacterial properties and a value of glucose detection limit of 2.29 mL, lower than the level in both healthy and diabetic people.

**Figure 1:**
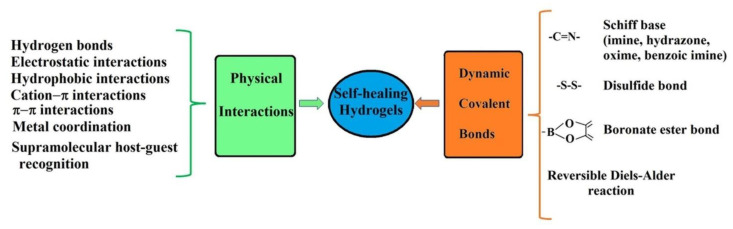
Physical and covalent bonds that provide the gels with self-healing ability.

Investigations into self-healable hydrogels have intensified to find durable materials and stable over time for biomedical applications [21,22,23,24,25,26,27,28,29]. Polymer hydrogels with shear-thinning and self-healing properties are suitable for their use in the fabrication of materials for tissue regeneration [30], cell encapsulation and delivery [31], 3D-priting for bone tissue engineering applications [32], drug delivery devices [33], in vivo applications [34], biosensors [35], etc. Recently, Qin and coworkers [36] reviewed, in a compressed form, the synthesis methods, self-healing mechanisms, and applications of conductive hydrogels reported in recent years. Their self-healing capacity is given by the existence in the polymer network of dynamic reversible bonds, either physical non-covalent bonds (such as hydrogen bonding) or covalent bonds, which can be restored after their destruction in certain physiological conditions [37,38,39]. Besides the hydrogen bonding, there are other types of physical interactions in the hydrogel network structure which can be reversibly broken and rebuilt under particular conditions of light, pH, or temperature: electrostatic [40], hydrophobic [41], host-guest supramolecular [42,43], cation–π [44] and π–π stacking [45] interactions, or metal coordination [46,47] (Figure 1).

The usual treatment for diabetics consists in the administration of insulin delivered by injections with syringes or microneedles or by oral administration. The continuous monitoring of the body glucose level and the appropriate dosage of administered insulin can prevent complications due to an overdose or a too small amount of medication. There are three main ways to continuously monitor glucose in the body: (1) the use of compounds containing borate groups (phenylboronic acid and its derivatives) with high specificity for cis-diols of glucose molecules; (2) the use of glucose oxidase that catalyzes the oxidation of glucose to hydrogen peroxide and gluconic acid; (3) the use of concanavalin A (Con A) with specific affinity to glucose [48]. This article reviews the recent progress in the preparation methods of the glucose-responsive insulin delivery systems based on hydrogels containing phenylboronic acid moieties. Moreover, the self-healing capability of these hydrogels is discussed. Finally, the perspectives and challenges for the fabrication of glucose-sensitive sensors, including those with boronate groups, are also discussed to highlight future research directions.

## 2. Hydrogels with Dynamic Covalent Bonds

Hydrogels containing dynamic covalent bonds had a rapid development in recent years due to their availability to be applied in biotechnology and medicine [27,28,29,30,49]. The existence of covalent bonds in these hydrogels gives them more stability and, the reversible breaking and restoring them take place much slower than for physical bonds [50].

**Figure 2:**
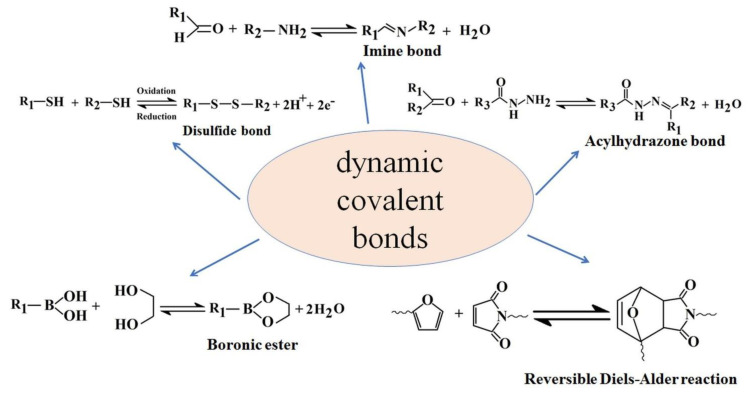
Illustration of the main dynamic covalent bonds.

Hydrogel materials containing dynamic Schiff base linkages have reversible covalent bonds which can self-repair and recover their structures after disruption. Schiff base materials are obtained by condensation reaction between amine groups and aldehyde groups obtaining a dynamic covalent imine bond (imine, hydrazone, or oxime) (Figure 2). Schiff base hydrogels are good candidates for applications in regenerative medicine, tissue engineering, drug delivery, wound healing, bioprinting, and biosensors, due to pH sensitivity of the imine bond from their structure [24]. 

Generally, imines exhibit good biocompatibility and, under physiological conditions, a rapid transition of the imine groups to their hydrolyzed form occurs [39]. Many hydrogel materials with applications in the biomedical field are based on biocompatible natural polymers (chitosan, collagen, etc.), whose amino groups can easily react with carbonyl groups by a nucleophilic addition under physiological conditions, leading to the formation of labile imine bonds in an acidic environment but stable at basic pH. Biomedical applications require the use of materials with a degree of toxicity as low as possible and, in this context, researchers focused on the use of a natural crosslinker for natural polymers.

Thereby, various natural monoaldehydes were used in order to obtain chitosan hydrogels with possible applications in medicine: citral [51], salicylaldehyde [52], pyridoxal 5-phosphate (the active form of vitamin B6) [53]. The reversible nature of the imine bond favors self-ordering of chitosan chains, leading to a layered structure with high stability. Hydrogels based on chitosan and nitrosalicylaldehyde with properties suitable for local cancer therapy were obtained by dynamic covalent chemistry [54]. Imination and transimination reactions are responsible for the formation of ordered clusters which represent the crosslinking nodes in the chitosan network structure. Considering their hydrolytic stability, oximes have the highest stability, followed by hydrazones and imines [55]. Self-healing hydrogels with potential applicability as a drug delivery system, with a similar structure to imine but with greater stability, were obtained by the reaction between acylhydrazine and aldehydes or ketones [56].

The ability of disulfide bonds to respond to different stimuli (pH, temperature, light, redox agents) was exploited by researchers to design hydrogels with self-healing behavior and/or degradable for medical applications [57]. Disulfide bonds, obtained by the reaction between two thiol groups, previously oxidized, represent important structural elements of proteins and peptides, with an essential role in the basic biological processes [58,59]. 

Self-healing hydrogels based on dextran [60], pectin/chitosan [61], cellulose/poly(ethylene glycol) were designed based on the thermo-reversibility of the Diels–Alder reaction between a conjugated diene and a dienophile [62]. 

The combination of various preparation methods allows the development of hydrogels with improved properties, expanding their medical applications area. Thereby, hydrogels with a double crosslinked network, based on adipic dihydrazide and sodium hyaluronate with furylamine groups, were prepared by combining the Diels–Alder click reaction with the aldehyde-amine Schiff base reaction [63]. These hydrogels have proven to be excellent candidates in scaffolds tailoring for cartilage tissue engineering. 

Smart hydrogels able to respond to biological stimuli for advanced functional applications in medicine can be designed by including boronate ester groups with dynamic character into the biocompatible natural polymers [64]. Chemical/physical crosslinked iminoboronate-chitosan hydrogels, with antimicrobial activity given by the presence of boric acid residue, were prepared from chitosan by using 2-formylphenylboronic acid as a crosslinker [65]. The hydrophobic/hydrophilic segregation of aromatic iminoboronate groups with hydrophobic properties and of hydrophilic chitosan is responsible for the formation of 3D supramolecular architectures [66].

In the current context, when diabetes is the third cause of mortality in the world, the finding of new self-healing materials sensitive to glucose and able to release specific drugs represents a priority for researchers. The boronate ester bond, obtained by the condensation reaction between boronic acid or its derivatives and 1,2-diols or 1,3-diols, has attracted attention due to, on the one hand, its reversible formation and, on the other hand, its glucose sensitivity [67]. 

## 3. Glucose-Sensitive Boronate Ester Group 

The current treatment of diabetes mellitus, which supposes patients self-injection with insulin, has the disadvantage of limited accuracy in blood glucose control and frequent injection. Knowing the capacity of phenylboronic acid (PBA) to bind with diols, some self-regulated insulin delivery systems with sensitivity to glucose under physiological pH and temperature were developed to eliminate the inconveniences mentioned above [68]. Phenylboronic acid (PBA) is preferred in the design of glucose-sensitive sensors due to its main advantages: durable affinity for glucose in physiological conditions, insulin release simultaneously with glucose detection, sensitivity to the changing pH and glucose level, possibility to modify its chemical properties by introducing various substituent groups, its non-toxicity, high stability [69]. The ability to interact with diols from other molecules than glucose and in vivo degradation represent the disadvantages which limit the PBA use. The preparation of such systems based on polymers containing PBA groups and the evaluation of their glucose responsiveness were reported firstly by Shiino and coworkers [70]. They demonstrated that the gels containing a completely synthetic polymer can be used in the design of some insulin delivery systems, replacing the glucose-sensitive proteins used until then. Insulin is released by a mechanism that involves the competition between the binding insulin and higher affinity of glucose molecules for PBA moieties. 

**Figure 3:**
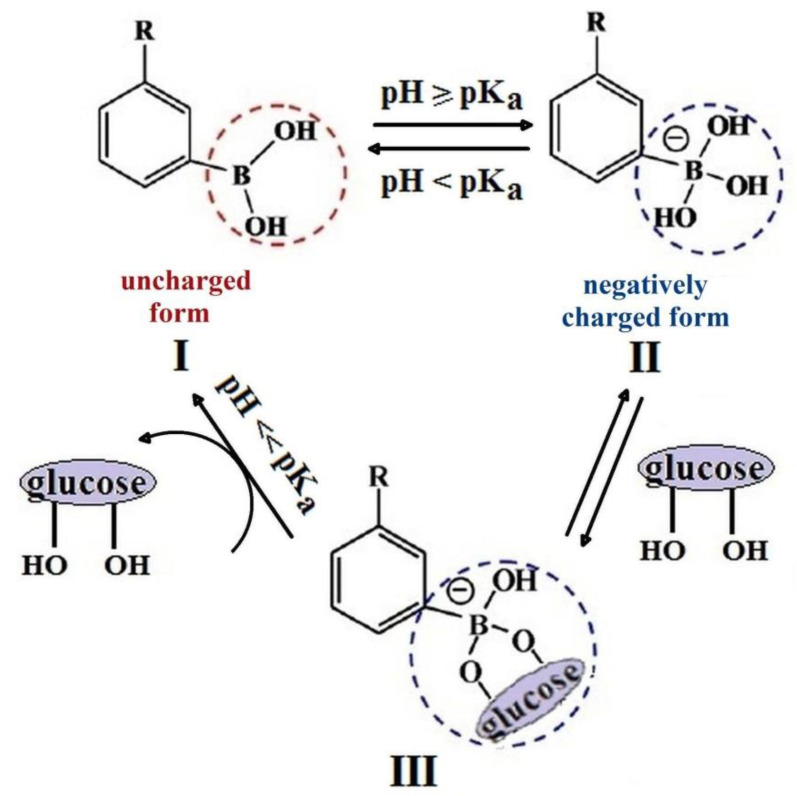
Configurations of PBA in water and complexation equilibrium between PBA-derivatives and glucose.

PBA-derivatives can adopt two forms in water which are in equilibrium: a trigonal-planar configuration (neutral and hydrophobic form) (I) and a tetragonal configuration (negatively charged hydrophilic form) (II) (Figure 3). For a pH value higher than pKa of PBA, the II forms (negative) are preponderant and, at a pH below pKa, most PBA moieties are in I form (neutral). By adding glucose to the system, the negatively charged form (II) binds with glucose, giving the III complex and more hydrophobic uncharged form, I, converts to the hydrophilic charged one. Depending on the substituent nature, the pK_a_ values of PBA derivatives were reported as varying between 7 and 9 (about 8.8 for PBA) [71]. 

Under physiological pH condition (about 7.4), the number of negatively charged configurations, responsible for the glucose complexation, is small due to the low acidity of PBA. The attachment to the phenyl ring of a strongly electron-withdrawing substituent determines the impoverishment of the boron atom in electrons, making the PBA moiety more acidic (pK_a_ lower than 8.8) and consequently, an increase in the number of species able to complex when glucose takes place. Moreover, the glucose-response rate increases by introducing electron-withdrawing substituents to the phenyl ring [72]. The acidity of PBA depends on the nature, size, and position of the substitute of the phenyl ring. Knowing that the introduction of an electron-withdrawing substituent into a compound causes the increase of its hydrophobicity, limiting its use in aqueous environments, the researchers focused on the introduction of such groups in combinations that would not affect the solubility in water. 

The ability of derivatives containing PBA to change from a hydrophobic form to hydrophilic one by adjusting the pH and diol concentration was used for the development of different systems with application in the detection and the treatment of diabetes (for example, glucose sensors, insulin delivery systems), such as gels, micelles, capsules [73,74].

## 4. Phenylboronic Acid-Based Glucose-Sensitive Hydrogels

**Figure 4:**
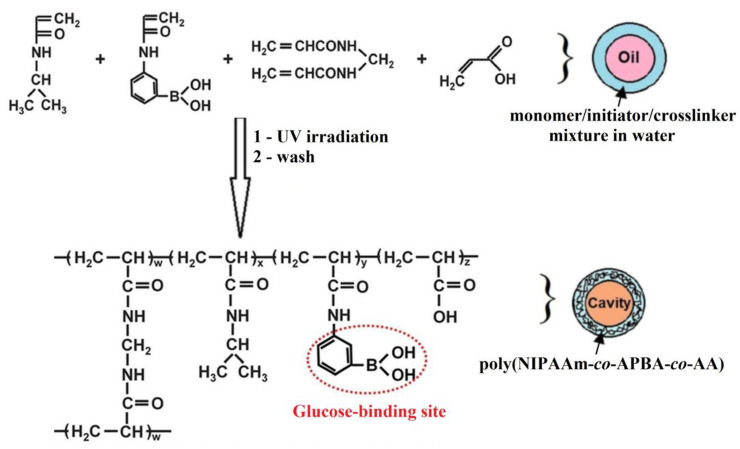
Preparation of poly(NIPAAm-*co*-APBA-*co*-AA) microcapsules (Adapted from reference [75] with permission from the Royal Society of Chemistry).

Poly(*N*-isopropylacrylamide) (poly(NIPAAm)) is known as a thermo-responsive polymer which exhibits a volume phase transition near body temperature. By increasing the temperature, at about 32 °C, poly(NIPAAm) undergoes reversible volume phase transition from the swollen hydrated state to the shrunken dehydrated state. The incorporation of glucose-responsive hydrophobic PBA into the poly(NIPAAm) chain determines the decrease of the volume phase transition temperature below 32 °C. In order to achieve the maximum swelling/shrinking volume transition at 37 °C in response to the changes of glucose concentration, Zhang et al. [75] included an amount of hydrophilic acrylic acid (AA) and 3-aminophenylboronic acid (APBA) in the copolymer containing poly(NIPAAm) (Figure 4). 

Poly(NIPAAm-*co*-APBA-*co*-AA) microcapsules obtained with 2.4 mol% AA showed reversible and repeated swelling and shrinking response at 37 °C for the changes of glucose concentration in blood in the range 0.4–4.5 g/L. In the medium with pH close to pK_a_ of the APBA sequence (pK_a_ = 8.75 [76]), the glucose-responsive microcapsules are in shrunken form at 37 °C. By increasing the glucose concentration, the charged PBA forms a complex with glucose, the equilibrium between uncharged–charged forms of PBA shifts toward charged hydrophilic phenylborate ions and the swelling of the microcapsules at 37 °C occurs. The decrease of glucose concentration induces the decomposition of the PBA-glucose complex, and the microcapsules shrink [75].

**Figure 5:**
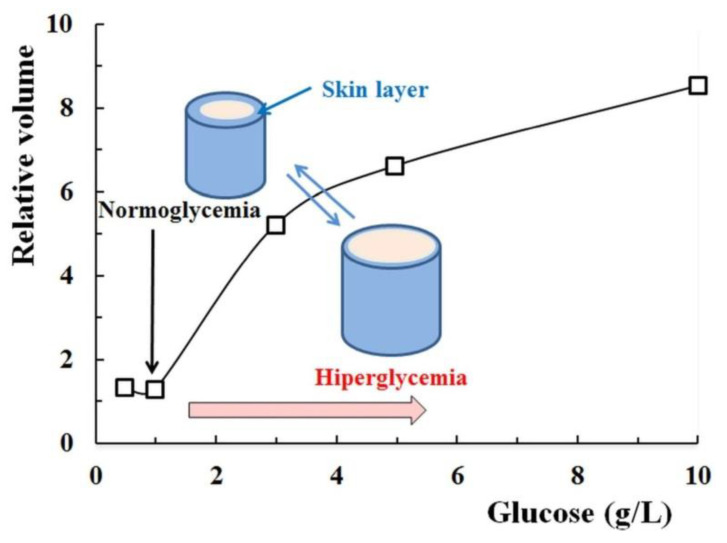
Volume changes of poly(NIPMAAm-*co*-AAmECFPBA) gel as a function of glucose amount at 37 °C (Adapted with permission from [77]. Copyright © 2023 WILEY-VCH Verlag GmbH & Co. KgaA, Weinheim, Germany).

Matsumoto et al. [77] synthesized a derivative of 4-(2-acrylamidoethylcarbamoyl)-3-fluorophenylboronic acid (AAmECFPBA) with *para*-carbamoyl and *meta*-fluoro substituents, which was then copolymerized with *N*-isopropylmethacrylamide (NIPMAAm) in order to obtain a gel with sensitivity to glucose presence. The pK_a_ value of the AAmECFPBA derivative containing the two substituents was around 7.2, suggesting good glucose-sensitivity at physiological pH. The optimum molar ratio between NIPMAAm and AAmECFPBA to obtain poly(NIPMAAm-*co*-AAmECFPBA) gel with the glucose-sensitivity at the physiological temperature and glucose concentration up to 1 g/L (normoglycemic value) was established as being 92.5/7.5. The gel containing the monomers in optimal ratio shrinks at pH = 7.4 by increasing the temperature due to the thermo-sensitivity of the poly(*N*-isopropylmethacrylamide) sequence. At a constant temperature, the gel is shrunken for the glucose concentration corresponding to normoglycemic value (1 g/L) and it starts to swell above this concentration due to the increase of pheneylboronate anions fraction (Figure 5). 

The shrinking process of poly(NIPMAAm-*co*-AAmECFPBA) gel as the glucose concentration decreases can be explained by the following processes: (1) a skin layer of collapsed (dehydrated) polymer is formed as a result of the dissociation of the phenylborate-glucose complexes; (2) the thickness of the collapsed layer increases, diminishing the permeability of the glucose molecules and the gel reveals an apparently constant volume; (3) the gel starts to shrink, reaching the completely shrunken state (overall collapsed phase) [78]. As a result of these processes, in poly(NIPMAAm-*co*-AAmECFPBA) gel, coexist two phases: one hydrated, inside the gel and the other, dehydrated, which can suppress the permeation of any preloaded molecules, for example, insulin.

Hydrogels with potential application in treatment of diabetes, due to their glucose-responsiveness, were prepared by simply mixing of the solutions of poly((ethylene oxide)-*b*-(vinyl alcohol)) (PEO-*b*-PVA) copolymer, α-cyclodextrin (α-CD), and PBA-terminated PEO as crosslinker [79]. For the hydrogel formation are responsible, on the one hand, the dynamic covalent bonds which can be established between PVA and PBA, and, on the other hand, the inclusion complexation between PEO and α-CD. The hydrogel showed a response for both glucose and fructose at a pH of 7.4 but the glucose concentration was of 30 g/L, much larger than that normal in blood (the approximate range of 0.7–1 g/L). The investigations on these hydrogels evidenced that the optimization of the glucose-responsiveness properties could be realized by increasing PVA and PBA content into the hydrogel. Sugita et al. [80] reported the preparation of a fluorescent chemosensor for *D*-glucose with extremely high selectivity, based on the supramolecular complex formed by encapsulation of an anthracene-based compound with a boronic acid moiety into the cyclodextrin cavity of fluorophenylboronic acid-appended β-cyclodextrin.

Poly(*N*-isopropylacrylamide-dextran-3-acrylamidophenylboronic acid) injectable nanogels (poly(NIPAAm-Dex-AAmPBA)), with good sensitivity to glucose under physiological conditions, were prepared by Wu et al. [81]. The nanogel containing the highest amount of Dex exhibits efficiency of insulin encapsulation and load capacity of 80.6% and 16.2%, respectively. Insulin-loaded poly(NIPAAm-Dex-AAmPBA) nanogels have reduced the glucose level in the blood of diabetic rats, keeping it at 51% of the baseline level for about 2 h.

**Figure 6:**
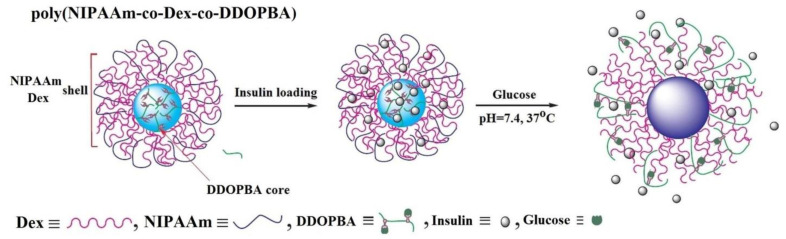
Schematic illustration of poly(NIPAAm-*co*-DEX-*co*-DDOPBA) nanogel as insulin delivery system (Adapted with permission from [82]. Copyright © 2023 Elsevier Ltd. All rights reserved).

The insertion of 4-(1,6-dioxo-2,5-diaza-7-oxamyl) phenylboronic acid (DDOPBA) into gels based on NIPAAm and Dex-grafted maleic acid induces glucose sensitivity in physiological pH conditions (Figure 6) [82].

In addition, the presence of NIPAAm gives the volume phase transition temperature, which shifts to higher values with the increase of acrylamide derivate and glucose content in the gel structure. At temperatures and pH values lower than the volume phase transition temperature and pKa of PBA sequence, respectively, a core-shell structure is formed. The core is formed by PBA moiety due to its hydrophobic character and the shell is constituted by hydrophilic NIPAAm and Dex (Figure 6). In presence of glucose and under a physiological environment (37 °C and pH = 7.4), the size of the core-shell structure of insulin-loaded nanogels changes. This change in size results from the protonation of PBA groups and their binding to glucose, leading to the increase of hydrophilicity which determines the gel swelling and the release of insulin.

**Figure 7:**
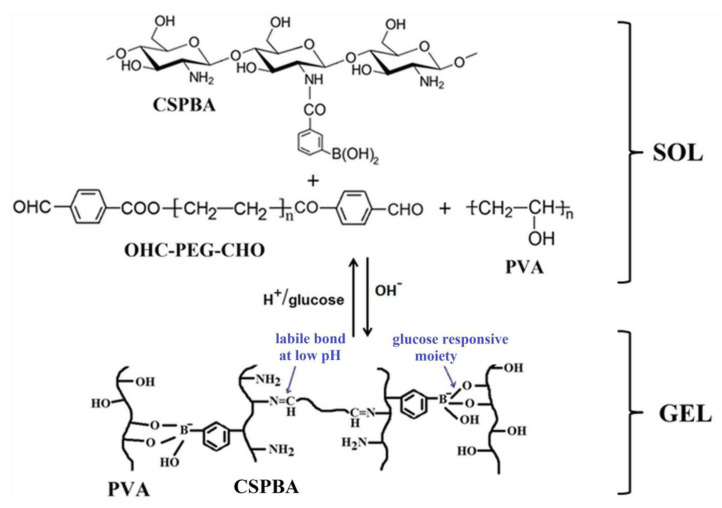
Structure of pH- and glucose-responsive CSPBA/PVA/OHC-PEG-CHO hydrogel (Adapted with permission from [83]. Copyright © 2023, American Chemical Society).

A major challenge for researchers is to design smart devices (“fully synthetic pancreas”) able to sense the high blood glucose level and to respond by releasing the appropriate dose of insulin. Hydrogels in which were encapsulated a protein drug (insulin) and live cells (L929) were developed as a medicated system for diabetic wound healing due to their capacity to promote neovascularization and collagen deposition [83]. These hydrogels were prepared by incorporation of insulin and L929 during the crosslinking process of a pH-responsive benzoic-imine and a glucose-responsive phenylboronate ester using phenylboronic-modified chitosan (CSPBA), PVA, and benzaldehyde-capped poly(ethylene glycol) (OHC-PEG-CHO) (Figure 7). 

This hydrogel showed reversible sol-gel transition at pH = 7 and, by adding of a small quantity of glucose solution, the gel-sol transition occurs. The decrease of pH or the increase of the glucose level determined more quickly the release of insulin from the hydrogel due to the swelling of the hydrogel network in a more acidic medium and the preference of phenylboronic groups to react with the hydroxyl groups from glucose. Thereby, the insulin release increased from 39% at pH 7.4 to 80% at pH 6.4 after releasing for 34 h. Moreover, the release of insulin increased from 9% for 1.75% CSPBA and 1% OHC-PEG-CHO to 39% for 1.5% CSPBA and 0.75% OHC-PEG-CHO, at pH 7.4 and release time of 34 h.

Glucose-sensitive spherical nanoparticles with a diameter of about 120 nm were obtained by self-assembly of poly(*D*-gluconamidoethylmethacrylate-*co*-3-methacrylamidophenylboronic acid) (poly(GAMA-*co*-MAPBA)) [84]. The insulin loading capacity into these nanoparticles was about 15% and its release increased by increasing the glucose level in the medium. The good cytocompatibility, proved by performing cell viability tests, suggested that these copolymers could be used in biomedical fields.

Recently, an organogel characterized by fast self-healing and responsive to three stimuli (pH, glucose, and redox-state) was prepared from PVA, 4-formylphenylboronic acid (4-FPBA), and 3,3′-dithiobis(propionohydrazide) (DPH) by dynamic covalent chemistry crosslinking reaction [85]. The responsiveness and self-healing property of these organogels are due to the break of dynamic covalent bonds and their ability to restore. By adding about 5 mg glucose, the boronate ester bonds between 4-FPBA and PVA dissociate, the network structure is destroyed, and the gel-sol transition occurs.

**Figure 8:**
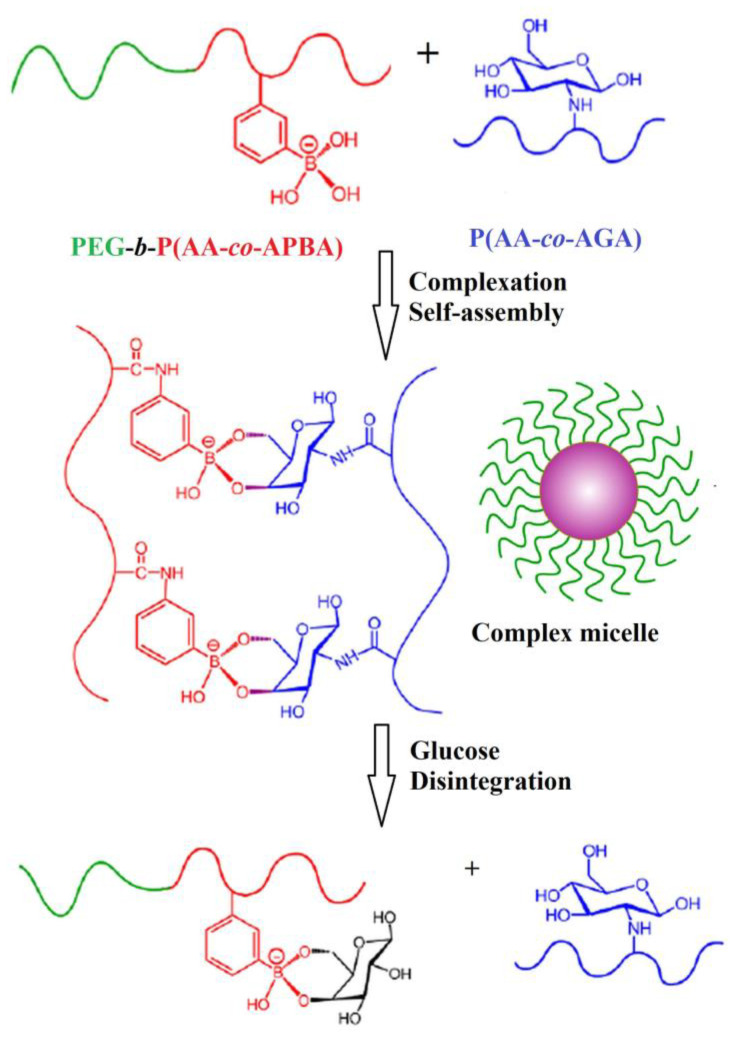
Schematic representation of the formation PEG-*b*-poly(AA-*co*-APBA/poly(AA-*co*-AGA micelles and their response in the glucose presence (Adapted with permission from [86]. Copyright © 2023, American Chemical Society).

Micelles based on PBA, which dissociate in the presence of glucose in physiological conditions, were obtained by self-assembling of poly(ethylene glycol)-*b*-poly(acrylic acid-*co*-3-aminophenylboronic acid) (PEG-*b*-poly(AA-*co*-APBA)) and poly(acrylic acid-*co*- acrylglucosamine) (poly(AA-*co*-AGA)) copolymers [86]. The micellization occurs due to the covalent bonds established between PBA and glycosyl (Figure 8). 

The core consists of the poly(AA-*co*-APBA)/poly(AA-*co*-AGA) complex and the shell is formed by PEG chains. The increase of poly(AA-*co*-AGA) content into micelles causes the decrease of the sensitivity to glucose. The highest sensitivity to glucose, in physiological conditions, showed the micelles with the PEG-*b*-poly(AA-*co*-APBA)/poly(AA-*co*-AGA) ratio of 1/0.75 (*w/w*).

The microgels based on PBA and *N*-alkylacrylamide derivatives can respond differently as the glucose concentration increases due to the different mechanisms of its binding to the boronate receptor [87]. Thereby, the microgels swell or shrink if the mechanism supposes the binding of glucose to a single or two boronate groups, respectively. Depending on the behavior in the presence of glucose, these microgels can be used as delivery for drugs (those that swell) and as a glucose sensor or as nanovalves included in an insulin delivery device (those that shrink). By choosing the appropriate experimental conditions, one of the two types of microgels can be obtained. The formation microgels which shrink as a response to glucose presence is favored if the following conditions are achieved: (i) initial microgel is swollen before adding glucose; (ii) the electrostatic repulsions between boronate groups are reduced by immobilization of positive charges on polymer chains; (iii) pH is above the pK_a_ value of the boronate, leading to an increase of the density of boronate groups, which favors the complexation of one glucose molecule with two boronates. 

Cambre et al. [88] prepared block copolymers based on poly(3-acrylamidophenylboronic acid) (poly(AAmPBA)) and poly(*N*,*N*-dimethylacrylamide) (poly(DMAAm)), able to self-assembly in solution and to form aggregates sensible to sugar presence. The dissociation of the aggregates as a response to the change of sugar concentration is dependent on polymer content and pH. These aggregates are able to encapsulate a hydrophobic component, which is released at high values of pH and sugar concentration. A sensor capable of continuously monitoring intravascular glucose based on a hydrogel containing poly(NIPAAm) and poly(AAmPBA) which can be attached to a stent was developed by Beier et al. [89].

**Figure 9:**
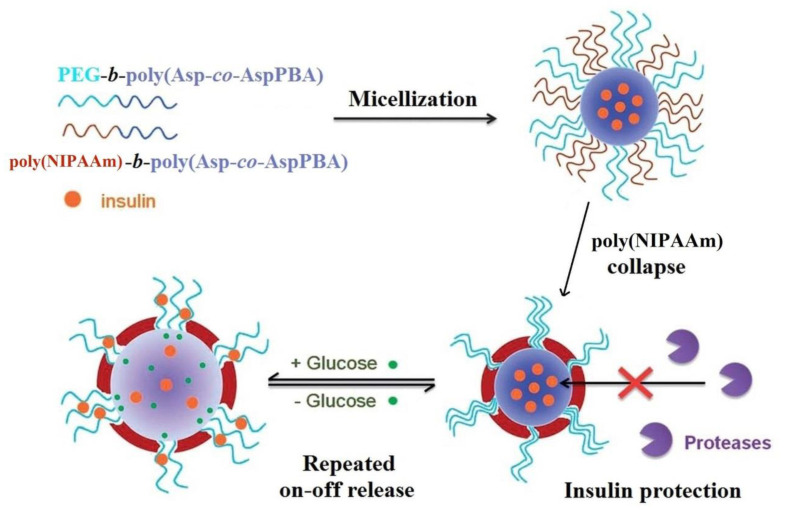
Formation of glucose-responsiveness polymeric micelles based on two diblock copolymers which exhibit a reversible swelling in response to the change of glucose concentration and insulin protection under physiological conditions (Adapted from reference [90] with permission from the Royal Society of Chemistry).

Polymeric micelles, with response to changes of the glucose concentration, by the self-assembly of poly(ethylene glycol)-*b*-poly(aspartic acid-*co*-aspartamidophenylboronic acid) (PEG-*b*-poly(Asp-*co*-AspAmPBA)) and poly(*N*-isopropylacrylamide)-*b*-poly(aspartic acid-*co*-aspartamidophenylboronic acid) (poly(NIPAAm)-*b*-poly(Asp-*co*-AspAmPBA)) were prepared [90]. For the poly(NIPAAm)/PEG ratio of 6/4 (*w/w*), complex micelles with poly(Asp-*co*-AspAmPBA) as the core and poly(NIPAAm), PEG as the hybrid shell were obtained (Figure 9). Generally, the most designed micelles present a biodegradable core, without the protection of loaded insulin, and in vivo enzymatic degradation occurs, reducing the effectiveness of insulin release by the system.

The protection of loaded insulin from the micelles core was provided by including glucose-responsiveness systems of poly(NIPAAm) moieties which, by their collapse, ensures the formation on the core surface of a protective membrane against enzymatic attacks. The hydrophobic interactions are responsible, on the one hand, for the insulin encapsulation into the micelles core, and, on the other hand, they can cause the denaturation of insulin [91]. The micelles swell reversibly in the presence of glucose, allowing the repeated on–off release of insulin (as a function of glucose level) due to expansion of the PEG channels embedded in the membrane formed by collapsed poly(NIPAAm). Cui and coworkers [92] synthesized complex micelles containing NIPAAm and AAmPBA sequences with a diameter of about 80 nm at pH of 7.4. These nanoparticles, which have shown thermo-, pH-, and glucose sensitivity, are promising materials for fabrication of insulin delivery devices.

The enhancement of the insulin loading content and the efficacy was realized by including into the micelles core of nitrilotriacetic acid (NTA) groups which, in the presence of Zn (II), determine the specific coordination between NTA-chelated Zn(II) and the histidine imidazole of insulin. Thereby, the glucose-responsiveness complex systems were obtained by micellization of poly(ethylene glycol)-*b*-poly(aspartic acid-*co*-aspartamidophenylboronic acid) (PEG-*b*-poly(Asp-*co*-AspAmPBA)) with poly(aspartic acid-*co*-aspartglucosamine-*co*-aspartnitrilotriacetic acid) (poly(Asp-*co*-AspGA-*co*-AspNTA[Zn])) [93] or poly(NIPAAm)-*b*-poly(Asp-*co*-AspGA-*co*-AspNTA[Zn]) [94]. Poly(NIPAAm) forms a continuous membrane around the core, which is glucose-responsive, providing effective protection of the insulin encapsulated in the micelles against protease degradation.

Some non-ionic spherical vesicular aggregates (named “niosomes”) based on cholesterol-based PBA-functionalized amphiphile can exhibit, in the presence of glucose, the changes in their morphological and physical-chemical properties [95]. The niosomes dissolve by adding glucose, due to the formation of the reversible boronate-diol complex. This behavior was employed in the development of vehicles capable of releasing insulin which, encapsulated, does not affect the stability of the self-assembly. The insulin is entrapped in the niosomes and, in the presence of glucose, the niosomal encapsulation is destroyed, releasing the drug.

**Figure 10:**
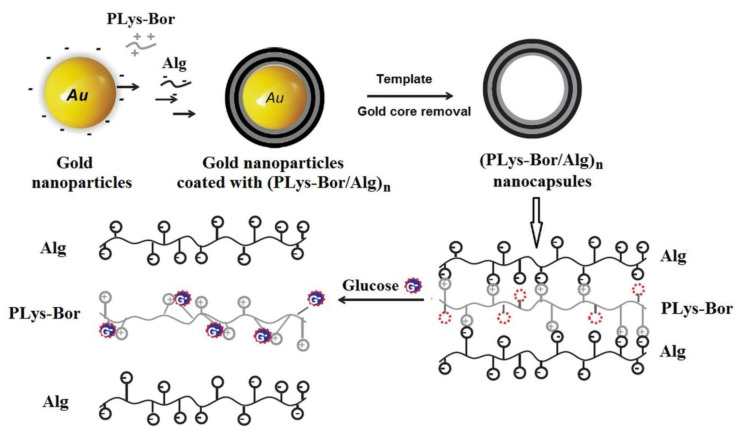
Illustration of the preparation strategy of (PLys-Bor/Alg)_n_ nanocapsules and the disassembly of the multilayers in the presence of glucose (Adapted with permission from [96]. Copyright © 2023 Elsevier Ltd. All rights reserved).

Recently, a new material based on phenylboronic-modified poly(lysine) (PLys-Bor) derivative and alginate (Alg) was developed by combining the templating method with the electrostatic-bonded layer-by-layer technique [96]. In the first step, a multilayer film was deposed onto a gold spherical substrate, and then, the gold core was removed via cyanide etching in order to obtain the nanocapsules sensible to glucose due to the presence of the boronic moieties attached to the poly(lysine) (PLys) chains (Figure 10).

The multilayer system is formed due to the electrostatic interactions between ammonium and carboxylate groups of PLys (polycation) and Alg (polyanion), respectively. The number of borate moieties starts to increase, and to interact with PLys derivatives by the addition of glucose. Three phenomena occur simultaneously: (1) new complexes are formed as a result of the electrostatic interactions between glucose and borate groups; (2) the glucose/borate complexes interact with ammonium groups from PLys and (3) PLys derivatives–Alg electrostatic interactions are destroyed, affecting the stability of the multilayer film. The system containing the PLys-Bor derivative has detected the glucose at concentration of about 0.5 g/L. The challenge for researchers refers to finding the solutions to the development of the glucose-responsive micelles loaded with insulin which can protect insulin from enzymatic degradation. 

**Figure 11:**
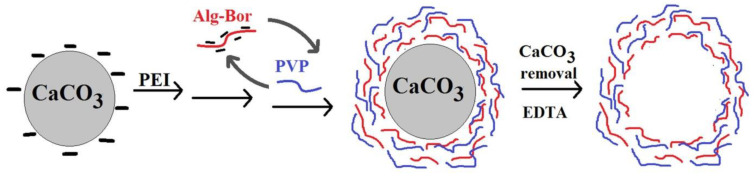
Elaboration of the glucose-responsive microcapsules based on AlgPBA and PVP (Adapted with permission from [97]. Copyright © 2023 Elsevier B.V. All rights reserved).

The templating and layer-by-layer methods were also combined to prepare the hollow microspheres based on poly(vinylpyrrolidone) (PVP) and an Alg derivative containing PBA sequences (AlgPBA) using the microparticles of calcium carbonate (CaCO_3_) as a removable core (Figure 11) [97].

Onto CaCO_3_ microparticles, pretreated with poly(ethylenimine) (PEI), was firstly deposited AlgPBA which at pH = 2 is adsorbed by hydrogen bonds and, at pH = 8, the electrostatic interactions between AlgPBA polyanion and PEI polycation are responsible for adsorption. Then, applying the hydrogen-bonded layer-by-layer technique based on the interactions between a hydrogen bond acceptor (the carbonyl groups of PVP) and a hydrogen bond donor (the boronic and carboxylic acid groups of AlgPBA in the function of pH), a PVP/AlgPBA multilayer shell was built. Finally, the CaCO_3_ core is removed by using ethylenediaminetetraacetic acid (EDTA) in order to obtain the hollow microspheres. The PBA moieties from the multilayer film cause the disassembly/destruction of the microspheres shell in the presence of glucose. These microcapsules exhibit dual functionality, being able to be loaded with drugs both in the core due to the CaCO_3_ porosity and in the shell due to the interactions that can be established between the drug and the carboxylate group according to the pH. This capacity of PVP/AlgPBA microspheres could be used both in the development of glucose sensors and in the treatment of diabetes by encapsulation of insulin (theranostic applications). 

**Figure 12:**
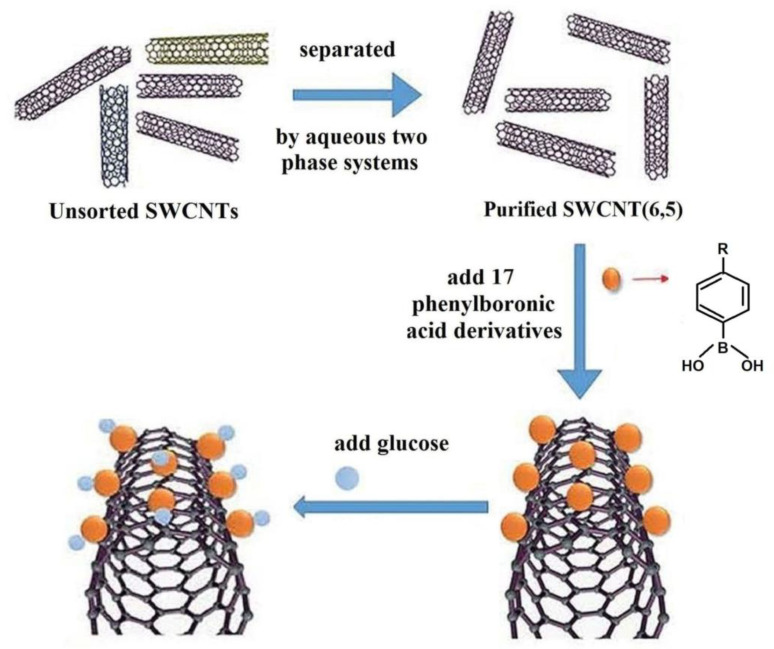
Illustration of SWCNTs preparation and glucose-sensitive complex formation. (Reprinted from reference [98] with permission from the Royal Society of Chemistry).

New glucose sensor material based on single-wall carbon nanotubes (SWCNTs) with strong fluorescence in near-infrared (NIR) was developed by Qiao et al. [98] binding the PBA derivatives by non-covalent bonds to the side walls of nanotubes, as shown in Figure 12. 

Glucose detection is based on the change of the photoluminescence intensity of SWCNT as a result of the binding of the hydroxyl groups from PBA moieties, attached to the nanotubes walls, with diols from glucose. 

The designing of plasmonic sensors has gained the attention of researchers in recent decades, considering the possibility of their exploration for the development of glucose-sensitive sensors. The activity of plasmonic sensors is based on the changing light properties after its interaction with metals or nanostructures containing metals [18]. Guo et al. [99] developed a plasmonic glucose sensor consisting of an optical fiber fabricated from composites of gold nanoparticles and hydrogels sensible to glucose. The gold nanoparticles, modified with carboxylic acid, were covalently immobilized onto the hydrogel matrix (based on 3-(acrylamido)phenylboronic acid (3-APBA)) through *N*-(3-dimethylaminopropyl)-*N′*-ethylcarbodiimide hydrochloride conjugation. The sensor showed high selectivity toward glucose and its responses were reversible and reproductible at various glucose levels.

As it is known, in type II diabetes, the pancreatic beta cells, which produce insulin for blood glucose to pass to cells, are exhausted due to the high glucose level in the blood. When the hemoglobin from blood is exposed for a long time to a high level of glucose, a derivative of hemoglobin is formed, glycosylated hemoglobin. The measurements of glycosylated hemoglobin from blood are the better method for diabetes detection. Knowing the capacity of PBA to form a complex with sugar, a biosensor was tailored by the coating of surface plasmon resonance gold chips with a nanofilm based on 4-vinylphenyl boronic acid (VPBA) [100]. The surface plasmon resonance sensor modified with VPBA received the signal at glycosylated hemoglobin (HbA1c) concentrations lower than the clinical concentration values, and the signal increased by increasing the HbA1c amount. The limit of detection was calculated as 2.86 μg/mL. The selectivity studies evidenced the neglectable interactions with IgG, hemoglobin, and human serum albumin from artificial plasma, and high affinity for HbA1c.

**Figure 13:**
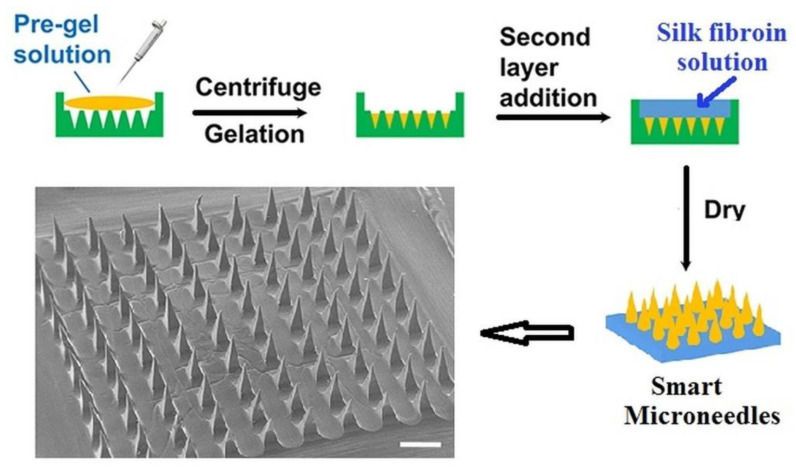
Preparation of microneedles by two-layer technique and their morphology determined at scale bar of 500 μm (Adapted with permission from [101]. Copyright © 2023, American Chemical Society).

As an alternative to the subcutaneous self-injection for insulin administration (usually used), a new non-invasive method which delivers transdermal insulin was reported by Chen et al. [101]. By using the two-layer technique, they designed a smart device consisting of a base of biocompatible silk fibroin (SF) and a needles region formed by a PBA/AAm hydrogel (semi-interpenetrating network) combined with SF (Figure 13). 

The sharp microneedles release insulin by controlling the glucose-sensitive skin layer formed on their surface by polymer collapse. These microneedles, which showed remarkable mechanical properties and stability remaining in the same shape after 7 days of keeping in an aqueous medium, could ensure a painless and a long-term supply of insulin as response to the increase of glucose in diabetic patients.

**Figure 14:**
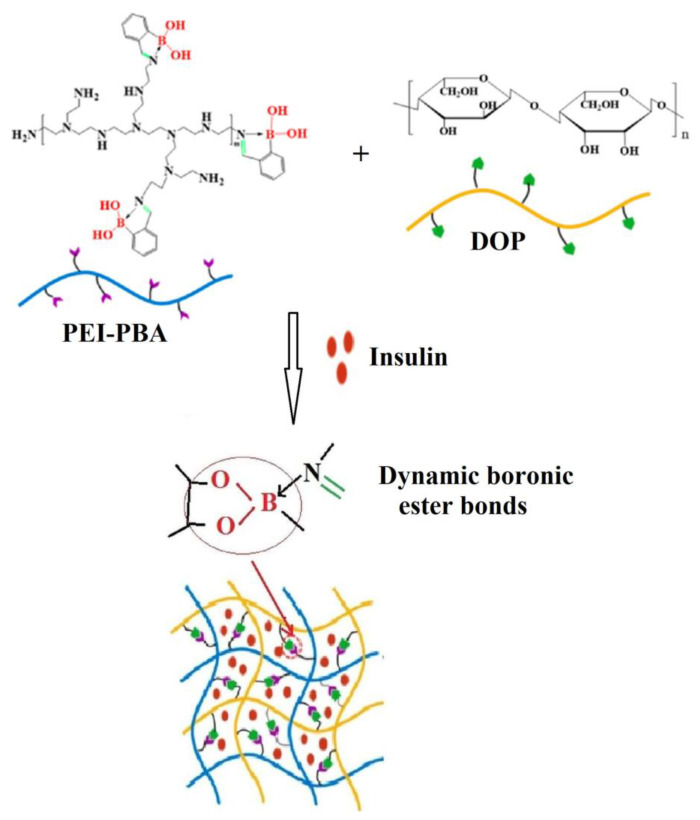
Schematic illustration of DOP/PEI-PBA hydrogel as insulin delivery system (Adapted from reference [102]. Copyright © 2023 by Liu, W. et al.).

Recently, Liu et al. [102] prepared a hydrogel based on the reaction at room temperature between 2-formylphenylboronic acid (2-FPBA), polyethyleneimine (PEI) containing the primary amine groups, and Dioscorea opposita Thunb polysaccharide (DOP) with cis-o-dihydroxy groups (Figure 14). The sensitivity of this hydrogel to pH and glucose, on the one hand, and its non-toxicity and biocompatibility, on the other hand, are characteristics that make it a good candidate in the design of a carrier with controlled release of insulin for the treatment of diabetes.

**Figure 15:**
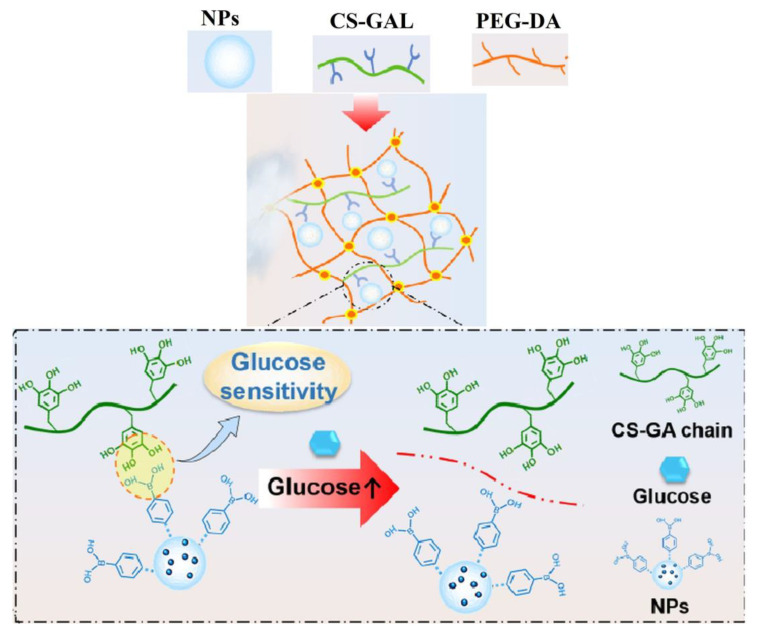
Preparation of PEG-DA/CS-GA/PEI-PBA hydrogel loaded with insulin nanoparticles (NPs) and its mechanism of glucose level regulation (Adapted with permission from [103]. Copyright © 2023, Tsinghua University Press).

Recently, a hydrogel for diabetic wound repair was developed by the incorporation of a sequence with antioxidant activity (gallic acid grafted onto chitosan, CS-GA) and one with hyperglycemic regulator property (polyethyleneimine modified with phenylboronic acid, PEI-PBA) into the poly(ethylene glycol)diacrylate (PEG-DA) network (Figure 15) [103]. 

The hydrogels changed the inflammatory microenvironment, accelerated angiogenesis, and diminished the wound inflammation, leading to healing after 20 days. Thereby, these hydrogels have proven to be effective materials for diabetic wound treatment.

**Figure 16:**
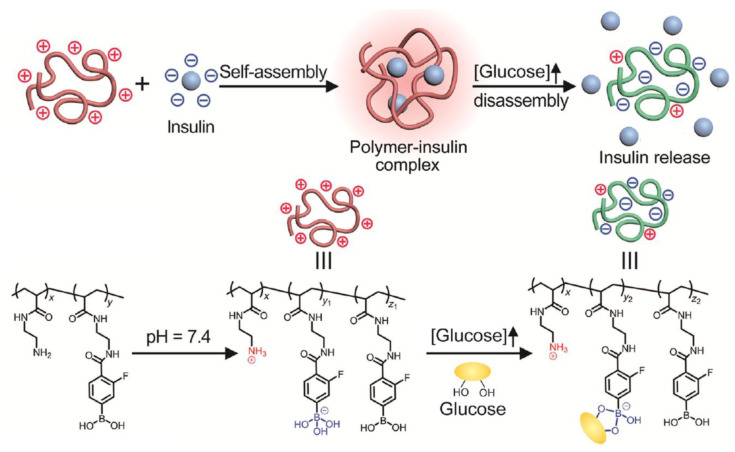
Schematic representation of polymer-insulin complex formation and of insulin release from complex under a hyperglycemic condition (Adapted from reference [104]. Copyright © 2023 by Wang, J. et al.).

A new strategy to design insulin-delivery devices as a response to glucose presence was proposed by Wang et al. [104]. This supposes the incorporation into an injectable gel matrix of a cationic polymer with pendant amino and PBA groups. This complex is positively charged at pH = 7.4, forming a stable suspension with high insulin loading efficiency (about 95%). Under hyperglycemic conditions, positive charge density decreases, due to the binding of glucose to polymer chains, and the electrostatic interactions between polymer and insulin decrease, facilitating insulin release (Figure 16). After returning to a normal blood sugar level, the decrease in positive charges no longer occurs and the rate of insulin release decreases.

Based on the capacity of PBA to interact with glucose, the next research trend refers to the designing of new medical devices which suppose a better adjustment of insulin delivery with glucose concentration from blood, finding less painful insulin therapy and easily available for administration.

## 5. Self-Healing Ability of Phenylboronic Acid-Based Hydrogels 

Research on the design of self-healing hydrogels with applicability in the biomedical field was intensified in recent years, knowing that any small defect in these materials can irreversibly affect their structure, making themunable to fulfill the role for which they were tailored. The pH sensitivity of diol-boronic acid complex gives the possibility to obtain the hydrogels able to completely recover their structure and their rheological properties (self-healing behavior). The restructuring mechanism, when the pH value changes, was discussed in detail in Section 2 of this review.

Hydrogels based on boronic acid, with self-healing property in an acidic and neutral environment, were prepared by Deng et al. [105] by using a strategy which supposes the mixing of boronic acid-containing copolymers (poly(2-acrylamidophenylboronic acid-*co*-*N*,*N*-dimethylacrylamide)) with diol-containing (co)polymers (poly(vinyl alcohol) or poly(dopamine acrylamide-*co*-*N*,*N*-dimethylacrylamide)). The boronic acid groups are capable of intramolecular coordination between oxygen from carbonyl groups and boron, leading to self-healing boronate ester hydrogels at pH ≤ 7. The hydrogels containing PVA exhibited higher strength compared to catechol crosslinked hydrogel (poly(dopamine acrylamide-*co*-*N*,*N*-dimethylacrylamide)), which is characterized by lower crosslinking density. The oxidation of the dopamine sequences determined the worsening of the self-healing capacity. 

**Figure 17:**
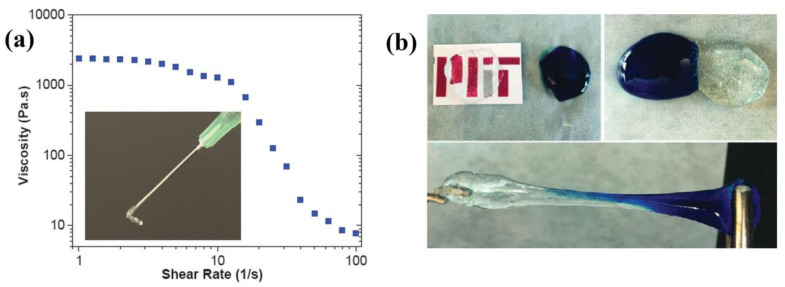
(**a**) Shear-thinning behavior and (**b**) exemplification of the self-healing property of the gel based on PEG and 3-fluorophenylboronic acid formed at pH = 7 (Reproduced with permission from [106]. Copyright © 2023 WILEY-VCH Verlag GmbH & Co. KGaA, Weinheim).

Hydrogels with potential applicability as 3D substrates for cells culture or as drug delivery systems were prepared by equimolar reaction between PEG with four arms terminated with PBA or its derivatives and PEG with four arms terminated with diol [106]. Hydrogels are not obtained at a pH lower than a pKa value corresponding to terminal PBA groups, while a rigid and brittle gel is obtained at a pH higher than pKa of terminal PBA groups. Thereby, the gels with great self-healing ability were obtained around the pKa value corresponding to terminal PBA groups (for example, pH of 7.8, 7.2, and 6.5–6.7 for terminal PBA, 3-fluorophenylboronic acid, and 2-formylphenylboronic acid, respectively). Due to the reversible and dynamic character of the formed boronic esters, these hydrogels based on PEG, formed at about pH = 7, are injectable (shear thinning properties, Figure 17a) and capable of recovering their structure after removing the applied stress (Figure 17b).

Knowing the reversibility of the PBA-glucose complex, an injectable hydrogel with self-healing ability was prepared based on the interaction between multiple PBA groups of polymer with multiple glucose units [107]. A shear thinning and self-healing hydrogel is formed using polymers containing 10–60% boronic acid groups in an equimolar ratio with glucose groups. The hydrogel network structure, broken by applying a high strain, is quickly recovered within a few seconds.

The multi-responsive hydrogels represent an important platform to design intelligent devices for applications, such as tissue regeneration/repair, drug delivery, sensors, bioinks, soft robotics, etc. [108]. One such hydrogel, containing the reversible dynamic boronate ester and disulfide bonds, was prepared by the crosslinking of cis-diols or catechol-containing hydrophilic polymers using a crosslinker based on boronic acid (bis(phenylboronic acid carbamoyl) cystamine) [109]. Dynamic covalent chemistry of boronate ester and disulfide bonds offers the possibility to use these hydrogels for designing the materials sensitive to pH changes, glucose presence, and redox-induced responsiveness. Moreover, the hydrogels reveal self-healing properties without the addition of a healing agent due to the ability to reorganize the complex between boronic acid groups and diols. The rheological properties can be tailored by adjusting the ratio between the acid-based crosslinker bis(phenylboronic acid carbamoyl) cystamine and the polymers containing cis-diols and catechol. Thereby, a larger amount of the acid-based crosslinker bis(phenylboronic acid carbamoyl) cystamine in the gel determines the increase of the crosslinking degree, leading to the enhancement of the rheological properties. The hydrogels based on boronic acid (*N*,*N*-dimethylacrylamide and pinacol protected ester of 2-acrylamidophenylboronic acid) and PVA proved to be suitable for cell culture media being able to be used as a support in dynamic co-cultures of human fibroblasts and breast cancer cells [110]. Benzoxaborole-catechol hydrogels, with excellent self-healing capacity and dual pH/glucose response, were obtained by the simple mixing of two copolymers containing benzoxaborole and catechol pendant groups [111].

**Figure 18:**
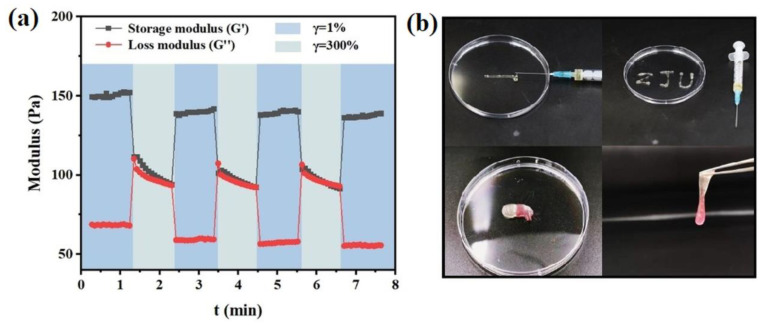
Illustration of (**a**) alternate-step strain test and (b) injection and self-healing phenomenon of the hydrogel based on 4-carboxy-3-fluorophenylboronic grafted chitooligosaccharides and guar gum (Adapted with permission from [112]. Copyright © 2023 Wiley-VCH GmbH).

Lu et al. [112] reported the synthesis of injectable glucose-responsive hydrogels with good self-healing capacity and biocompatibility by combination of 4-carboxy-3-fluorophenylboronic grafted chitooligosaccharides with guar gum. The self-healing property is given by the reversible phenylborate ester bonds established between the two components (Figure 18). 

This hydrogel can be taken into account in the development of insulin delivery carriers because, in the presence of glucose, 4-carboxy-3-fluorophenylboronic grafted chitooligosaccharides/guar gum hydrogel disintegrated, leading to two natural polymers with good biocompatibility and biodegradability.

Phenylboronate covalent chemistry was applied recently to obtain the dynamic natural photonic crystal hydrogels with great potential in designing devices for applications, such as chemo/biosensors for environment monitoring, anticounterfeiting, optical devices, energy conversion, and biomedical engineering [113]. The injectable ordered colloidal crystals, with self-healing capacity after physical damage, were obtained by the self-assembling of phenylboronic acid-based microgels in a glycomonomer solution. 

Recently, Xiang et al. [114] synthesized new dynamic and self-healing hydrogels by the crosslinking reaction between aryl boronates and diols groups attached to a poly(ethylene glycol) macromer with four arms. These hydrogels showed high affinity and specificity to glucose and low ability to bind to non-glucose sugars, proving to be good candidates for designing glucose sensors. 

## 6. Conclusions and Future Opportunities 

Phenylboronic acid-based hydrogels are materials with interesting properties that make them attractive for clinical applications in diabetes therapy. The present review summarized the recent advances in glucose responsive hydrogels which contain phenylboronic acid or its derivatives in their structure, with focus given to their response mechanism in the presence of glucose, the release of loaded insulin, the recent preparation techniques, and their self-healing mechanism. The inclusion of the reversible and dynamic boronate ester groups in various polymer structures and architectures allowed the design of new materials with properties adapted to the challenges in the biomedical field, such as support for cell culture, gene delivery, enzyme modulation, etc. 

The main challenge in the design of phenylboronic acid-based hydrogel is the finding of polymer network structures that afford the correction of the pH value of phenylboronic acid sequences (pKa = 8.68) to the physiological pH (near 7.4) maintaining the fast response to glucose. Another aspect that should be considered in the preparation of glucose-sensitive hydrogel refers to the decrease of its sensitivity to other sugars (for example, fructose, mannose, etc.) and the increase of selectivity for glucose. In addition to those mentioned above, the following aspects should be taken into account in future research: (1) the expanding of the architectures of the hydrogel network to improve its mechanical properties, preserving its self-healing properties, by diversifying the phenylboronic acid derivatives and diols used as starting compounds; (2) the development of new hydrogel materials that maintain their mechanical and self-healing properties for a longer use period; (3) the designing of phenylboronic acid-based systems with a faster and selective response to glucose, leading to the rapid release of loaded insulin in a dosage that ensures the maintenance of normal blood glucose level; (4) the finding of a less traumatic administration method compared to the injection that is currently used; (5) the biocompatible and easily degradable compounds in physiological microenvironments should be taken into account in the designing of glucose-responsive hydrogels; (6) the high potential that Chinese medicine monomers can have in treating diabetics should not be neglected [115]. 

Currently, the used glucose sensing technique, which is stressful and irritating for the patient, supposes the finger pricking and the collection of a blood drop. Therefore, the priority for researchers is to find a non-invasive or minimally invasive technique for glucose detection from body fluids, such as saliva, sweat, tears, urine, or interstitial fluids, that can be applied at the clinical level. In terms of effective systems, it is necessary to consider the composition which can ensure the higher selectivity for glucose compared to other sugars from the human body. In the design of a glucose-responsive sensor, a balance between the selectivity for glucose and its mechanical properties should be ensured. The new generation of biosensors involves the wearing of these devices by the patient either by attaching to the skin or implanting in the body. Therefore, biosensors should be flexible, elastic with self-healing properties to ensure their wearing comfort. Moreover, they must be biocompatible and non-toxic, so as not to worsen the patient’s condition. To fulfill these conditions, it is necessary to develop multifunctional and innovative devices whose operating principles are based on multiple mechanisms. The elaboration of the devices for continuous glucose monitoring and insulin delivery in real time remains also a challenge for scientists. The devices developed until now have significant deficiencies in terms of precision, accuracy, and stability. Despite the important number of studies on glucose-sensitive systems, many aspects related to their mechanical and self-healing properties, their biocompatibility and biodegradability under physiological conditions, as well as the effectiveness of insulin release to keep the glucose level within normal limits, still remain to be resolved.

## Data Availability

Not applicable.

## Abbreviations

AA	acrylic acid
AAmECFPBA	4-(2-acrylamidoethylcarbamoyl)-3-fluorophenylboronic acid
AAmPBA	3-acrylamidophenylboronic acid
AGA	acrylglucosamine
Alg	alginate
APBA	3-aminophenylboronic acid
Asp	aspartic acid
AspAmPBA	aspartamidophenylboronic acid
AspGA	aspartglucosamine
AspNTA	aspartnitrilotriacetic acid
α-CD	α-cyclodextrin
CS-GA	gallic acid grafted onto chitosan
CSPBA	phenylboronic-modified chitosan
DDOPBA	4-(1,6-dioxo-2,5-diaza-7-oxamyl) phenylboronic acid
Dex	dextran
DMAAm	*N,N*-dimethylacrylamide
DOP	*Dioscorea opposita* Thunb polysaccharide
DPH	3,3′-dithiobis(propionohydrazide)
EDTA	ethylenediaminetetraacetic acid
2-FPBA	2-formylphenylboronic acid
4-FPBA	4-formylphenylboronic acid
GAL	gallic acid
GAMA	*D*-gluconamidoethylmethacrylate
Lys	Lysine
MAPBA	3-methacrylamidophenylboronic acid
NIPAAm	*N*-isopropylacrylamide
NIPMAAm	*N*-isopropylmethacrylamide
NTA	nitrilotriacetic acid
OHC-PEG-CHO	benzaldehyde-capped poly(ethylene glycol)
PBA	phenylboronic acid
PEG	poly(ethylene glycol)
PEG-DA	poly(ethylene glycol)diacrylate
PEI	poly(ethylenimine)
PEO	poly(ethylene oxide)
PEO-*b*-PVA	poly((ethylene oxide)-*b*-(vinyl alcohol))
PLys	poly(lysine)
PLys-Bor	phenylboronic-modified poly(lysine)
PVA	poly(vinyl alcohol)
PVP	poly(vinylpyrrolidone)
VPBA	4-vinylphenyl boronic acid
